# The influence of biochar on the content of carbon and the chemical transformations of fallow and grassland humic acids

**DOI:** 10.1038/s41598-021-85239-w

**Published:** 2021-03-11

**Authors:** Marta Cybulak, Zofia Sokołowska, Patrycja Boguta

**Affiliations:** Institute of Agrophysics, Polish Academy of Sciences, Doświadczalna 4, 20-290 Lublin, Poland

**Keywords:** Ecology, Environmental sciences, Biochemistry, Environmental chemistry, Physical chemistry

## Abstract

There is limited information regarding the effect of biochar (BioC) on the fertility of fallow and grassland soils, as well as on the properties of their humic acids (HAs). The objective of this study was to evaluate with a 3-year field experiment the influence of BioC on the organic matter (OM) in Haplic Luvisol. BioC (obtained via wood waste pyrolysis at 650 °C) was applied to the soil of subplots under fallow and grassland at doses of 0, 1, 2 and 3 kg m^−2^. The soil samples were collected eight times. The physicochemical properties were determined for the soil and BioC by analysing the density, pH, surface charge, ash, and organic carbon content. Based on the changes in the structure of the HAs and their quantity in the soils, the chemical properties of the HAs were determined. The maximum BioC dose caused an increase in the content of C_org_ and HAs. BioC did not influence the humification degree coefficients of the HAs originated from fallow, whereas in the grassland, there were significant changes observed in these coefficient values, indicating that BioC may stimulate and accelerate the humification process of soil HAs. Increasing the BioC doses caused an increase in the soil’s HA content, suggesting an increase in soil sorption capacity. The fluorescence data showed BioC addition to the soil caused an increase in the number of structures characterised by low molecular weight and a low degree of humification.

## Introduction

Unfavourable changes in the prices of fertilisers and agricultural products in the nineties contributed to the exclusion of some agricultural lands from crop production. In the history of global agriculture, fallows have been known for centuries as an integral aspect of crop rotation. Allowing farmland to fallow can effectively improve the self-rehabilitation of arable soil and solve agroecological problems such as biodiversity loss, soil fertility loss, and over-cultivation^1^. Nevertheless, long-term fallow cannot satisfy requirements at the local level, and moreover, it results in a strong decline in the ecosystem^1^. Modern agriculture is currently attempting to restore utility value to these soils, and fallow reclamation has become a goal in this context^2,3^. Moreover, agriculture is strongly evolving towards sustainable systems and organic farming, thus it is necessary to seek solutions that will not influence soil conditions in a negative manner (e.g. by chemical or physical degradation)^4^. The answer to this problem could be biochar (BioC)—a solid carbon-rich product generated during waste biomass pyrolysis in a strongly reduced oxygen atmosphere^5,6^, which is intended to improve the quality and fertility of soils. This distinguishes it from similar substances like charcoal, which is primarily intended for energy use. Research on the impact of BioC on the physicochemical properties of Haplic Luvisol soil showed that the addition of BioC to fallow and grassland influenced the dynamics of changes in the physiochemical parameters of the examined soils^4^.

There are many scientific reports on the effect of BioC addition to agriculturally cultivated soils on the physicochemical properties of the soils (mainly model studies)^7–10^. The literature also includes valuable studies on the effect of BioC on soil organic matter (OM), including the humic and fulvic acids of differently used soils or composting manures^11,12^. However, there is no information regarding the potential influence of BioC on improving the fertility of fallow soils, or on the chemical and sorption properties of their humic acids (HAs). According to the literature, BioC may provide a potential soil amendment approach. BioC, due to its high porosity, may improve water absorption, sorption capacity, and nutrient retention, as well as the sorption of organic and inorganic contaminants^6^. Moreover, BioC contains micro- and macroelements and has high stability in soil^9,13^. This component of BioCs plays an important role in determining their potential for environmental use. Clearly, the composition and distribution of soluble OM present in BioC vary significantly according to the type of feedstock, activation, and pyrolysis temperature^14,15^ as well as the pH and BioC particle size fraction^16^. The content of soluble OM in BioC correlates with the BioC volatile matter content and acid functional group density^17^. In previous study^15^, the soluble OM obtained from 10 BioC samples produced from different feedstocks was characterized using EEM/PARAFAC (excitation-emission matrix/ spectroscopy coupled with parallel factor) analysis and four fluorescent components: C1, a humic-like component; C2, a protein-/tannin-like component; C3, a fulvic acid-like component; and C4, a terrestrial humic-like component were identified. There are also available, interesting results of research that show that the content of dissolved organic carbon (DOC) extracted from BioC ranges from 435 to 5000 mg kg^−1^, and that high soluble OM release occurs under high temperature and alkaline environmental conditions^18^. The authors also identified, using fluorescence analysis, four HA-like substances (C1, C2, C4, and C5), one fulvic acid-like substance (C3), and one tryptophan-like substance (C6).

The addition of BioC could also have an effect on the pool and chemical properties of the OM in soils^19,20^, particularly on HAs. This fraction (HAs) plays a special role among OM compounds by affecting the physical^21^, chemical^22^, and biological^23^ properties of the soil. The acidic character and the nature of negative colloids make HA a valuable buffer compound in soil^24,25^ and one of the most highly charged substances among naturally occurring polyelectrolytes^26^. As a consequence, HAs are responsible for binding cations in the soil, and thus for the circulation and availability of micro- and macroelements. However, HAs are subject to constant structural changes in soil under the influence of various factors. These processes are still poorly understood, although they determine the fate of many other substances in the soil. An analysis of changes in the HA fraction is particularly interesting due to the wide spectrum of HA behaviour in soil ranging from that of fulvic acids to that of humins, i.e. depending on the environmental conditions, HAs are found in highly soluble forms similar to fulvic acids, or as poorly soluble compounds more similar to humins^27^. Moreover, the HAs in soils vary widely in structural and sorption properties, including their molecular weights (25,000–200,000 Da), and their number of functional groups, aromatic rings, and aliphatic chains^28,29^. Due to such high variability and sensitivity of the HAs to different conditions, it can be assumed that BioC supplementation to soil may also influence the chemical properties of soil HAs. These changes should be monitored, in particular the HA fraction, in relation to the nature of BioC and the method of its production. In the context of pyrolysis and the use of BioC in soil, the higher stability of HAs compared with, for example, fulvic acids, may be crucial. BioC could contain some HAs hidden in the pores such that BioC-derived HAs would increase the HA pool in the soil. This assumption is more likely for HAs than for fulvic acids whose thermal stability is lower (a significant part of the fulvic acids can be destroyed during BioC production). We based these assumptions on our previous studies of the thermal properties of HAs and fulvic acids^30^ in an N_2_ atmosphere. Those results showed that fulvic acids were degraded at lower temperatures due to their higher content of O-containing groups and lower content of aromatic structures. This also seems to justify the need to study the changes in HA fraction under the influence of BioC supplementation.

Further, due to insufficient fertilisation and crop production in monoculture, most agricultural mineral soils are characterised by a low content of OM and significant depletion of HAs. Such environments require additional supplementation with organic carbon compounds. According to different sources^31,32^, adding BioC to degraded soils may have significant potential for improving their fertility and productivity by enriching organic carbons, as well as for the protection of plants against diseases. For example, mixing BioCs with Acrisols at a 100 g kg^−1^ dose increased the pH from 4.9 to 8.7, and this resulted in a 15-fold increase in the DOC concentration (from 4.5 to 69 mg dm^−3^)^31^.

Soil degradation due to land mismanagement is a major global concern that threatens economic and rural development. Therefore, the aim of this work was to investigate the effect of BioC addition to degraded soils (fallow and grassland). This goal was realised by examining (1) the effect of BioC on the OM sorption properties of soils from fallow and grassland, and (2) BioC’s influence on the HAs of these soils as a function of BioC dose and the 3-year duration of the experiment. By examining the dynamics of changes in the content and properties of OM, and its most important fraction, HAs, we would like to examine the suitability of BioC to improve the quality of degraded soils.

## Results and discussion

### Physicochemical and chemical properties of soils and BioC

The physicochemical and chemical characteristics of the soils and BioC, as well as selected chemical properties of the HAs isolated from the soil and BioC are shown in Table 1.

**Table 1:** Physicochemical and chemical characteristics of soils, BioC and isolated HAs.

Material	Soil and BioC properties					HAs properties		
	d (g cm^−3^)	C_org_ (g kg^−1^)	A (g kg^−1^)	pH (H_2_O)	Q (cmol kg^−1^)	Q_HA_ (cmol kg^−1^)	E_2/6_	ΔlgK
Fallow	2.61	9.8	973	6.2	5.90	359	45	0.83
Grassland	2.60	10.2	967	6.7	7.10	344	48	0.86
BioC	1.46	154.0	432	8.3	107	172	10.6	0.54

*d* density, *A* ash content, *C*_*org*_ organic carbon of soil and BioC, *Q* surface charge of soil and BioC at pH 9.0, *Q*_*HA*_ surface charge of HAs at pH 9.0, *ΔlgK* HAs’ humification index (wavelength 400 and 600 nm), *E*_*2/6*_ HAs’ absorbance ratio (wavelength 465 and 665 nm).

The properties of soils and BioC, such as the d, C_org_, A, pH, and Q, were presented in detail previously^4^. Briefly, soils were characterised by a typical d value for mineral soils ≈ 2.60 g cm^−3^, and by a relatively low content of C_org_ and a high content of A. The pH of the soils was weakly acidic. The examined soils were characterised by low Q values, indicating a low content of organic structures dissociating to the negative surface charge (mainly carboxylic and phenolic groups). The HAs obtained from fallow and grassland were characterised by high Q_HA_ values (about 50 times higher in comparison with the Q values of fallow and grassland). The d value of BioC was typical for organic materials (1.46 g cm^−3^), moreover, the BioC contained a high content of OM, which was expressed as C_org_. The pH of BioC was alkaline. This material was also characterised by a high Q value, which indicated its favourable sorption properties.

The results of our studies showed that the E_2/6_ values were similar for the HAs originated from the two studied soils, suggesting a similar ratio of lignin-type compounds resistant to humification to the structures with a high humification degree. The ΔlgK reached values of 0.83 and 0.86 for HAs isolated from grassland and fallow, respectively, indicating a low degree of HA humification (Kumada’s classification for low humification degree of HAs: ΔlgK = 0.8–1.1)^33^. Slightly higher ΔlgK values obtained for the grassland HAs compared with the fallow suggested a higher content of less humified compounds, such as cellulose, hemicellulose, and lignin^34^.

The ΔlgK of HAs isolated from BioC reached a value of 0.54, suggesting the presence of highly humified compounds, in comparison with soil HAs (Kumada’s classification for high humification degree of HAs: ΔlgK < 0.6). A particularly low value for the E_2/6_ parameter of the HAs fraction isolated from BioC indicated a low content of lignin-type compounds^31^.

The BioC properties presented above largely depended on the conditions of the pyrolysis process, as well as on the feedstock. The BioC used in our experiment was obtained via wood waste (coniferous and deciduous) pyrolysis at a relatively high temperature of 650 °C. The first rapid stage of this process (to 200 °C) could be attributed to the evaporation of moisture and light volatiles, the second one (200–500 °C) to devolatilisation and decomposition of hemicelluloses and cellulose, and the last one to the degradation of lignin and other OM with stronger chemical bonds^35^. High temperatures (600 °C and higher) can result in a decrease in CEC (Cation-exchange capacity and the content of surface functional groups in the produced BioC^36^. Moreover, the final product may also show higher hydrophobicity^37^. Contrarily, a high temperature of pyrolysis can result in an increase in the content of carbonised fractions and facilitate thermal degradation of refractory compounds like cellulose and lignin. Moreover, high temperatures can lead to the release of higher amounts of volatile matter and to the formation of more pores^38^. The resulting product obtained under high temperature reveals greater surface area, which is a desirable feature of biomaterials introduced into soils to improve their aggregate structure and the sorption properties of their hydrophobic components or other soil molecules which can be effectively sorbed into micropores. Feedstock based on wood wastes was also crucial for achieving the desired properties of the analysed BioC. Woody biomass can be characterised by low moisture, high calorific value, and high bulk density^39^. Low-moisture biomass is favourable due to the reduction in the heat energy and time required for pyrolysis. The application of wood feedstock also resulted in a product with an increased specific surface area and a lower pH compared with non-wood derived BioCs^40^. Moreover, wood BioC contains a greater amount of humic substances than rice husk or bamboo BioCs^41^. In connection with the above, the BioC production technology used in our research, as well as its temperature, heating, and feedstock conditions, seemed to result in the production of BioC with interesting and desirable properties. Moreover, the process efficiency was also high, whereas the cost intensity was low: carbonising products were mainly BioC (65–80% of the input energy) and process gases (15–35% of the input energy), and the process was based on autothermal roasting without the use of additional energy, catalysts, or chemical additives. BioC produced from wood waste (FLUID technology) showed a qualitative increase in physical and chemical properties in comparison with untreated biomass in relation to the moisture, quantity of carbon element, volatile matter, and calorific value.

### Effect of BioC on organic carbon and organic functional groups in soils: comparison between fallow and grassland

**Figure 1:**
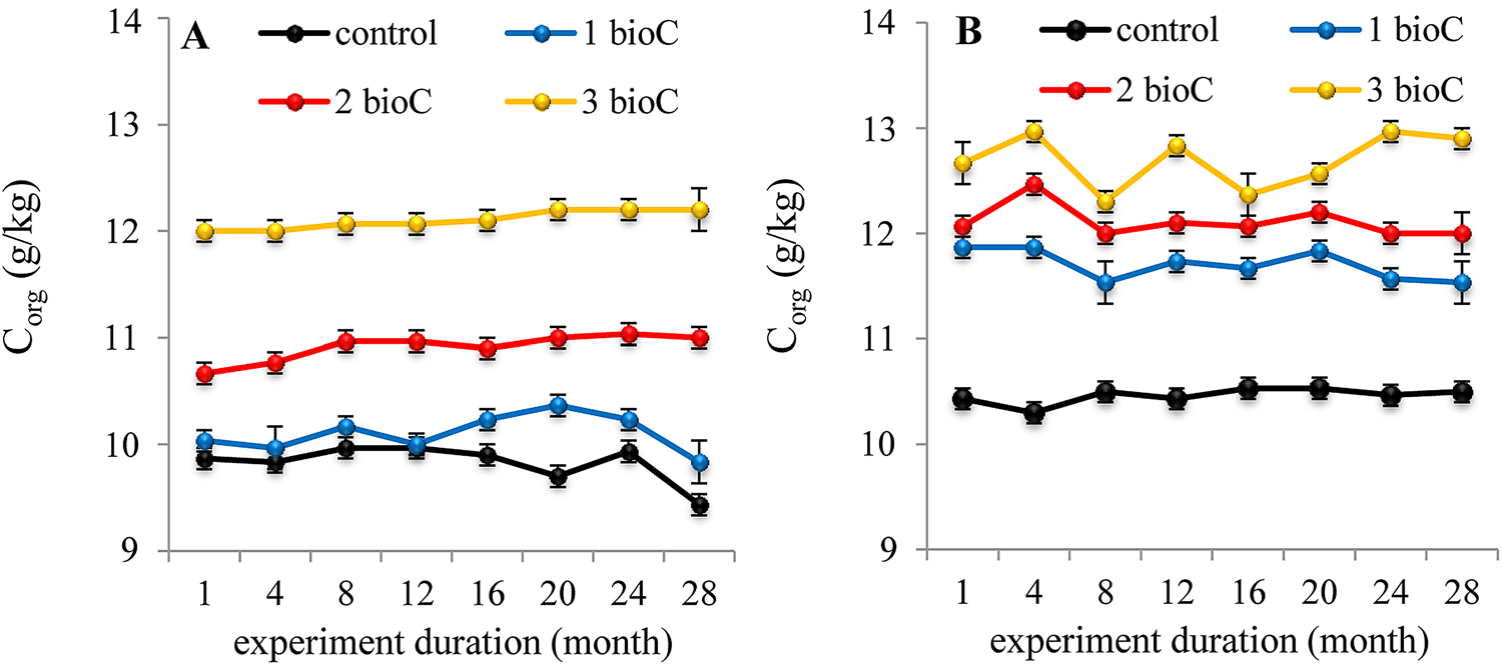
Changes in organic carbon (C_org_) content in soil samples collected from fallow (**A**) and grassland (**B**) amended with BioC (0, 1, 2, 3 kg m^−2^) as a function of time. Average values from 3 replicates in each term, ± standard deviation.

The addition of increasing BioC doses to fallow and grassland caused an increase in the C_org_ content of the soils (Fig. 1A,B). However, in case of the fallow, a significant increase was observed only for the two highest BioC doses, 2 and 3 kg m^−2^ (Fig. 1A), whereas in the case of the grassland, the lowest BioC addition also resulted in a significant increase in the value of the discussed parameter (Fig. 1B). Generally, the addition of maximum BioC dose caused an increase in C_org_ content which ranged for different months from 3 to 20% and from 15 to 25% as compared to control values in the fallow and grassland, respectively. So, we can assume that the highest BioC dose was optimal for soil enrichment in organic carbon.

After increasing the C_org_ content in soil samples from fallow and grassland, this parameter remained at an almost constant level throughout the entire 3-year experiment. It was probably related to the high stability of organic compounds that entered the soil with BioC. Different studies^11,42^ show that C_org_ released from BioC may remain stable in soil for millennia; moreover, the BioC obtained at high temperatures reveals a structure like graphite, which has a positive effect on the stability of the product^5^. Considerable stability of organic compounds contained in the BioC in relation to soil organic compounds could also result from the high content of divalent cations (Ca^2+^, Mg^2+^) forming insoluble salts with organic ligands of the BioC^26,43^.

The stability and chemical quality of the BioC used in our experiments also strongly depended on its content of volatile organic compounds. Their amount in the analysed BioC was approximately 150 g kg^−1^. This is a relatively low value, which resulted from the conditions of the pyrolysis process. High temperature of the process (650 °C) caused the breakdown of cellulose, hemicellulose, and lignin. As a consequence, the content of volatile matter markedly decreased, but the ash content increased. This was observed due to separation of volatile fractions into low-molecular-weight liquids and gases, which evaporated from the BioC^40^. Studies on the effects of pyrolysis temperature on the physicochemical properties of different BioCs^44^, showed that volatile matter ranged from 340 to 540 g kg^−1^ in the BioCs obtained at 300 °C as well as from 60 to 190 g kg^−1^ in BioCs produced at 600 °C. Because BioC lability was found to be strongly controlled by the relative amount of a more aliphatic and volatile component, the measurement of volatile compound content may be a convenient predictor of BioC carbon (C) longevity. The lower the level of volatile components, the more stable the BioC in the soil, and consequently, the longer the impact on soil properties^45^. This fact was confirmed in our research: the most significant changes were observed in the third year of the experiment. From an environmental point of view, the volatile matter content of the BioC is an important factor affecting its agronomic value as a soil amendment. Chars that are rich in volatile matter content (i.e. a typical barbecue charcoal) would not be good soil amendments because they overstimulate microbial activity or immobilise the nitrogen (N) available to plants in the short term. What is more, soil amended with BioC having high volatile matter content showed a dramatic decline in soil respiration^45^. The relatively low content of volatile compounds in the BioC used in our research seemed to be safer for the environment. The literature shows that the release of volatile compounds into water and soil can have adverse effects on plants and microorganisms^46,47^.

**Figure 2:**
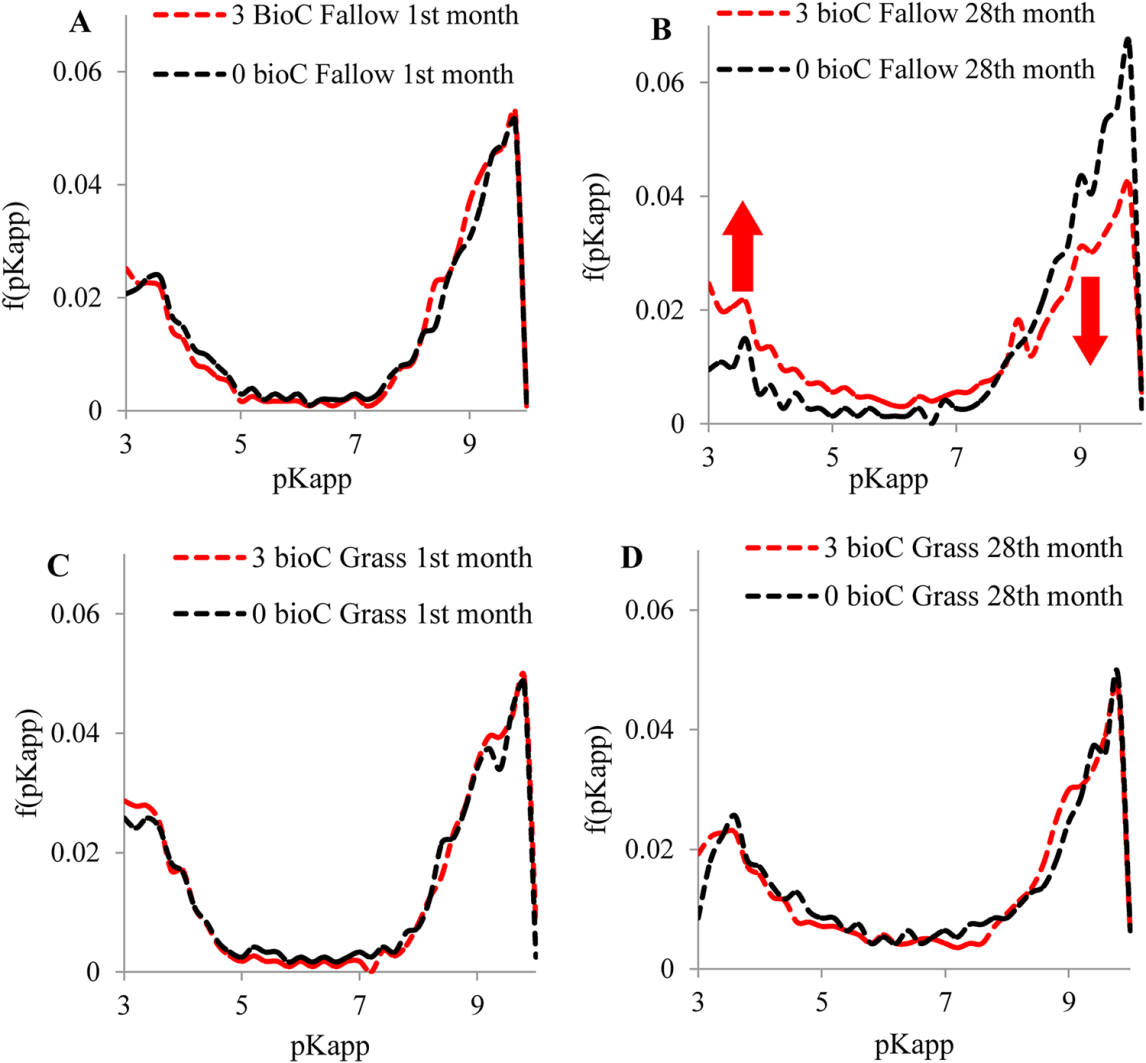
Normalized distribution function of dissociation constants of functional groups in fallow (**A**,**B**) and grassland (**C**,**D**) amended with BioC (0 and 3 kg m^−2^) in 1st and 28th month of field experiment.

The data obtained from potentiometric titration allowed for an estimation of the distribution functions of apparent surface dissociation constants in the fallow and grassland amended with the BioC. These characteristics illustrated quantitative and qualitative changes in surface functional groups. The exemplary distributions hare shown in Fig. 2A–D. The shape of the characteristics was similar for both fallow (Fig. 2A,B) and grassland (Fig. 2C,D), showing two main areas of functional group dissociation; the apparent dissociation constants observed at pKs from 3.0 to 5.0 were mainly attributed to strongly acidic carboxylic groups, whereas pKs occurring between 7.0 and 10.0 were primarily related to weakly acidic phenolic and hydroxylic groups originating mainly from organic structures and clay minerals^48,49^. The presence of the above structures determines both the sorption and buffer capacity of soil systems^48^. Surface functional groups dissociating at pKs 7.0–9.0 were dominant, revealing the higher content of phenolic and hydroxylic groups compared with carboxylic structures.

BioC application in the initial period after its addition to the soil (first month of experiment) did not cause significant changes in the apparent surface dissociation constants for either the fallow or the grassland (Fig. 2A,C). In the last year of the experiment (the 28th month of the experiment), a significant increase in the number of functional groups dissociating below pH 5.0 was observed for the fallow (e.g. carboxylic groups) in relation to the content of weakly acidic groups dissociating above pH 7.0 (according to the normalised distribution) (Fig. 2B). This fact indicates the long-term effect of BioC, particularly with respect to its structures that are rich in carboxylic groups. These conclusions are consistent also with the results of research on impact of a woody BioC on properties of a sandy loam soil and spring barley^50^, which demonstrated that BioC has a long-term effect, noticeable only a few years after its application to the soil. The effect of carboxylic groups of BioC is interesting because BioC production by biomass pyrolysis results in the partial degradation of oxygen-containing functional groups^51,52^. No similar changes were observed in the distribution of dissociation constants of grassland amended with BioC (Fig. 2D). This result is consistent with results of other studies^53^ which demonstrated no effect of BioC on the cation exchange capacity of soils under plant cover, a result which may be equivalent to the lack of BioC influence on the distribution of apparent dissociation constants in grassland. Changes in the quantitative relationships of functional groups observed in fallow at the 28th month probably occurred because of chemical transformations of the available pool of organic carbon compounds, as was suggested by the relatively constant amount of C_org_ throughout the duration of the experiment. Increasing the contribution of COOH groups may provide an important advantage in the agricultural context, because these groups dissociate at the pH typical of most soils, thus increasing the level of bound micronutrients.

### Influence of BioC amendment on quantity and acid–base properties of HAs in fallow and grassland

**Figure 3:**
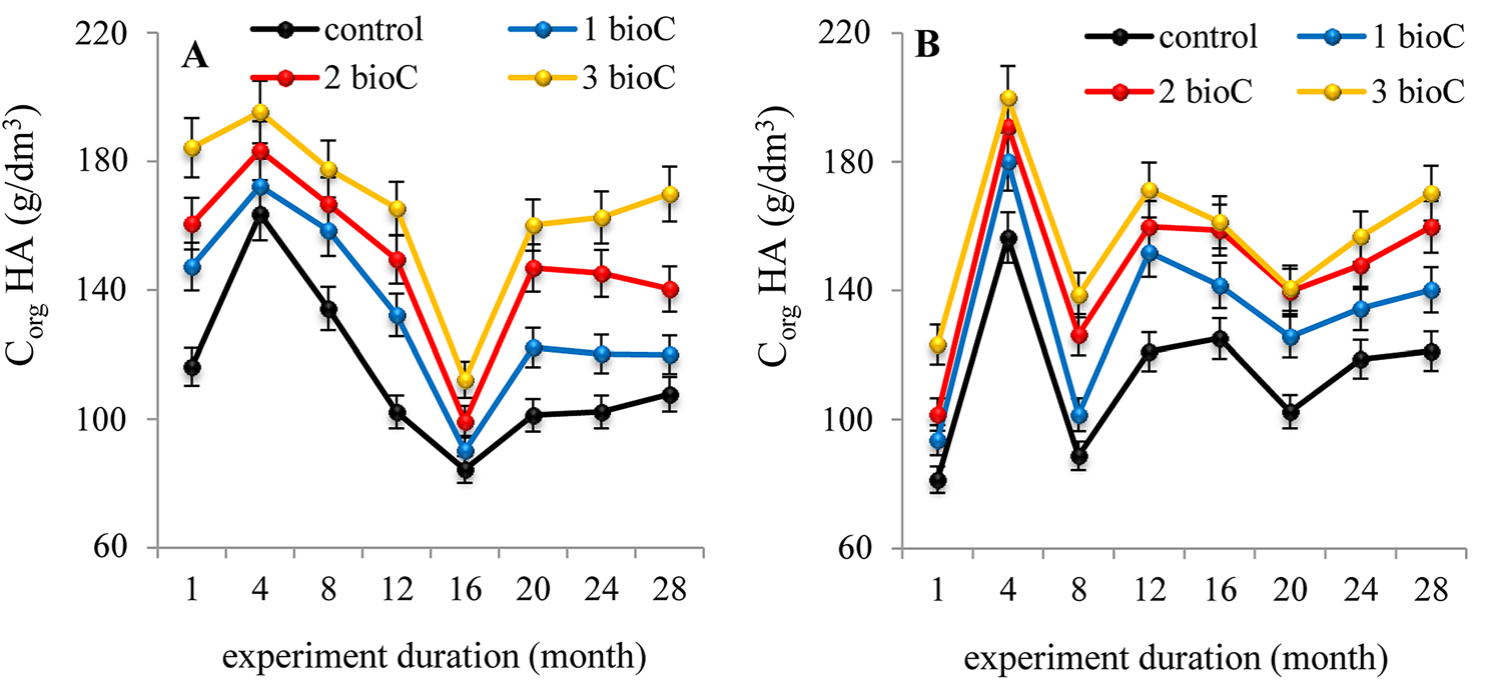
Changes in organic carbon concentration (C_orgHA_) in alkaline extracts of HAs isolated from fallow (**A**) and grassland (**B**) amended with BioC (0, 1, 2, 3 kg m^−2^) as a function of time. Average values from 3 replicates in each term, ± standard deviation.

The analyses of organic carbon concentration in the extracts of HAs (C_orgHA_) demonstrated that BioC amendment also influenced the total content of the HAs both in fallow and grassland. The influence of BioC doses on the content of HAs in the soils expressed as a concentration of organic carbon in the HAs extracts is shown in Fig. 3A,B. Increasing the doses of BioC caused an increase in the content of HAs in each month of the experiment, despite significant fluctuations in the content of HAs in the control soil (variations of C_orgHA_ in Fig. 3A,B). The content of soil HAs increased (after the addition of 3 kg m^−2^ BioC) by approximately 15%—both in fallow and in grassland. The observed decrease in the HA content in some terms most likely resulted from the intensified mineralisation process of native organic compounds^54^.

The increasing in the HA content with the increase in BioC dose was caused by the general increase in the level of organic compounds after adding BioC to the soil^42^. In addition, an increase in the HA content in the grassland could be the result of rotting plant residues and their transformation into simple humus compounds^54^. The intensity of changes in the HA content weakened in the last year of the experiment, indicating the stabilisation of their content in the soil^55,56^.

Another aspect of principal importance is the priming effect that BioC may exert on native soil organic carbon (SOC) and the converse, i.e., the priming effect of SOC on BioC C. This is important, especially if BioC is used for C storage capacity^57^. In our research, the priming effect was not measured directly, however from the changes in the C_org_ content and the C_orgHA_ content, we can conclude about positive priming effect. Moreover, the organic substances derived from BioC were characterised by relatively low mineralisation and high stability in the environment; this fact confirms the positive priming effect in our research. We showed that the wood waste BioC stimulated SOC mineralisation (positive priming)^58^. According to literature^57^, the positive priming from BioC may also be related to relatively greater lability of native organic C and its lower protection in soil with clay content < 200 g kg^−1^ (the clay content in Haplic Luvisol used in our experiment was approximately 70 g kg^−1^). The stabilisation of SOC and BioC C through organo-mineral associations is expected to be related to the soil clay content. In soils containing > 200 g kg^−1^ clay, stabilisation or decrease in native SOC mineralisation in the presence of BioC may occur through BioC induced organo-mineral interactions.

**Figure 4:**
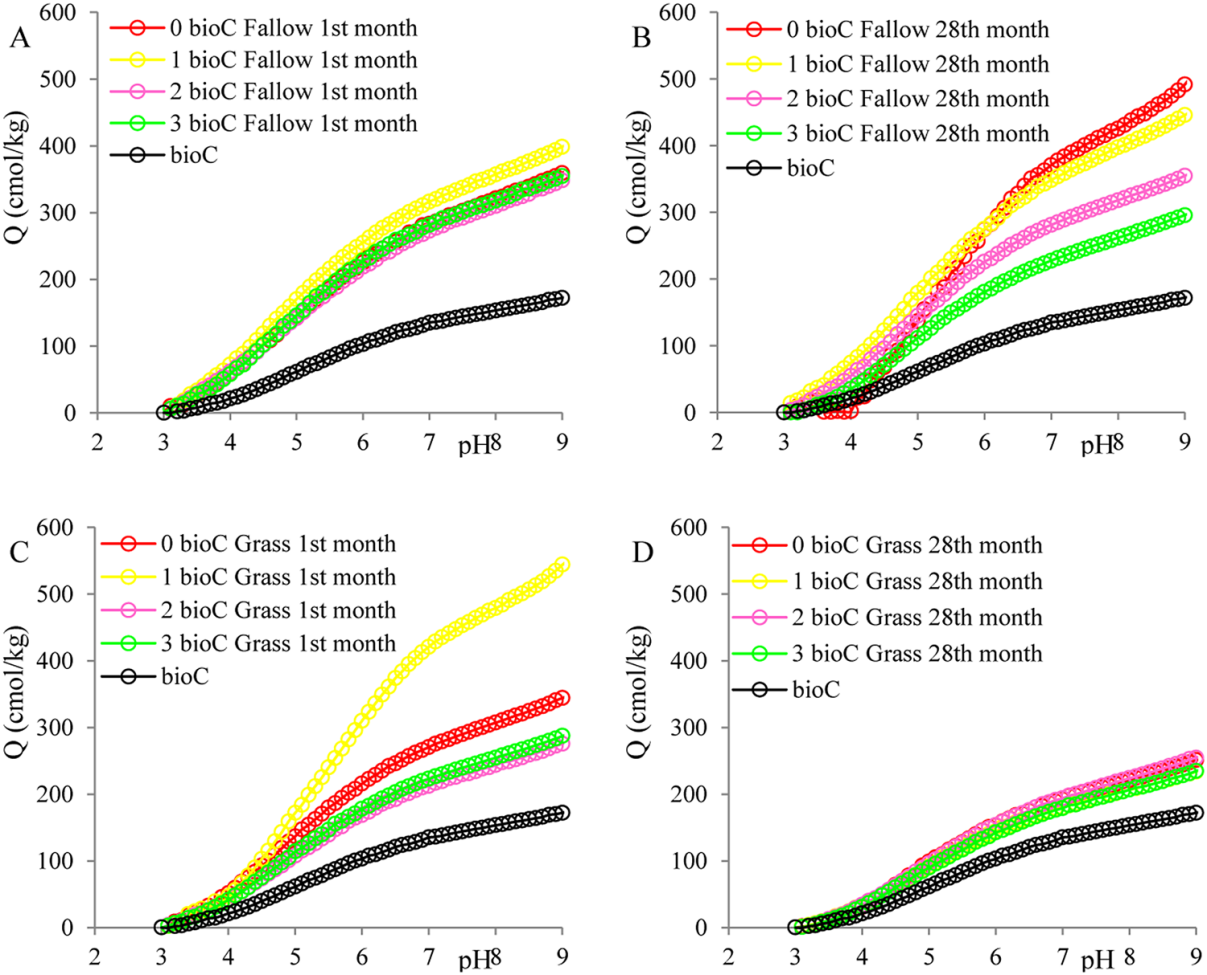
Dependence of surface negative charge (Q_HA_) on pH of the HAs solution. HAs obtained from fallow (**A**,**B**) and grassland (**C**,**D**) amended with BioC in 1st and 28th month of field experiment, as well as HAs obtained from BioC.

Data obtained from potentiometric titration allowed an estimation of the surface negative charge values in HAs (Q_HA_) obtained from fallow, grassland, and BioC in the pH range of 3.0–9.0 (Fig. 4A, Fig. 4B—fallow; Fig. 4C,D—grassland). The full dissociation of HAs occurs at pH > 8.0, above which the OH groups are deprotonated^26^, therefore we only report results in this pH range. Changes in the Q_HA_ values as a function of pH (Fig. 4A–D) were monotonic; these values increased towards an alkaline pH, which resulted from the fact that other fractions of functional groups dissociated successively at increasing pH values. Generally, in the first month of the experiment, the highest Q_HA_ values were observed for HAs obtained from fallow and grassland with the lowest BioC dose (Fig. 4A,C). This fact indicated that these HAs had the best sorption properties. In the last month of the experiment, the Q_HA_ values changed in an ambiguous way. The Q_HA_ at pH 9.0 values of HAs isolated from pure BioC were lower than those obtained from the soil, and moreover, BioC did not have an obvious effect on the Q_HA_ values of the soil HAs. Previous studies^4^ on impact of BioC on the physicochemical properties of Haplic Luvisol under different land uses, showed that BioC added to soil caused a significant increase in Q values in the last year of the experiment. Thus, we can conclude that BioC introduced OM with a variable surface charge but did not affect the soil’s Q_HA_. It is possible that the BioC doses used in our experiment were insufficient to raise the Q_HA_ values.

### Influence of BioC amendment on structure and chemical properties of HAs in fallow and grassland: spectroscopic approach

**Figure 5:**
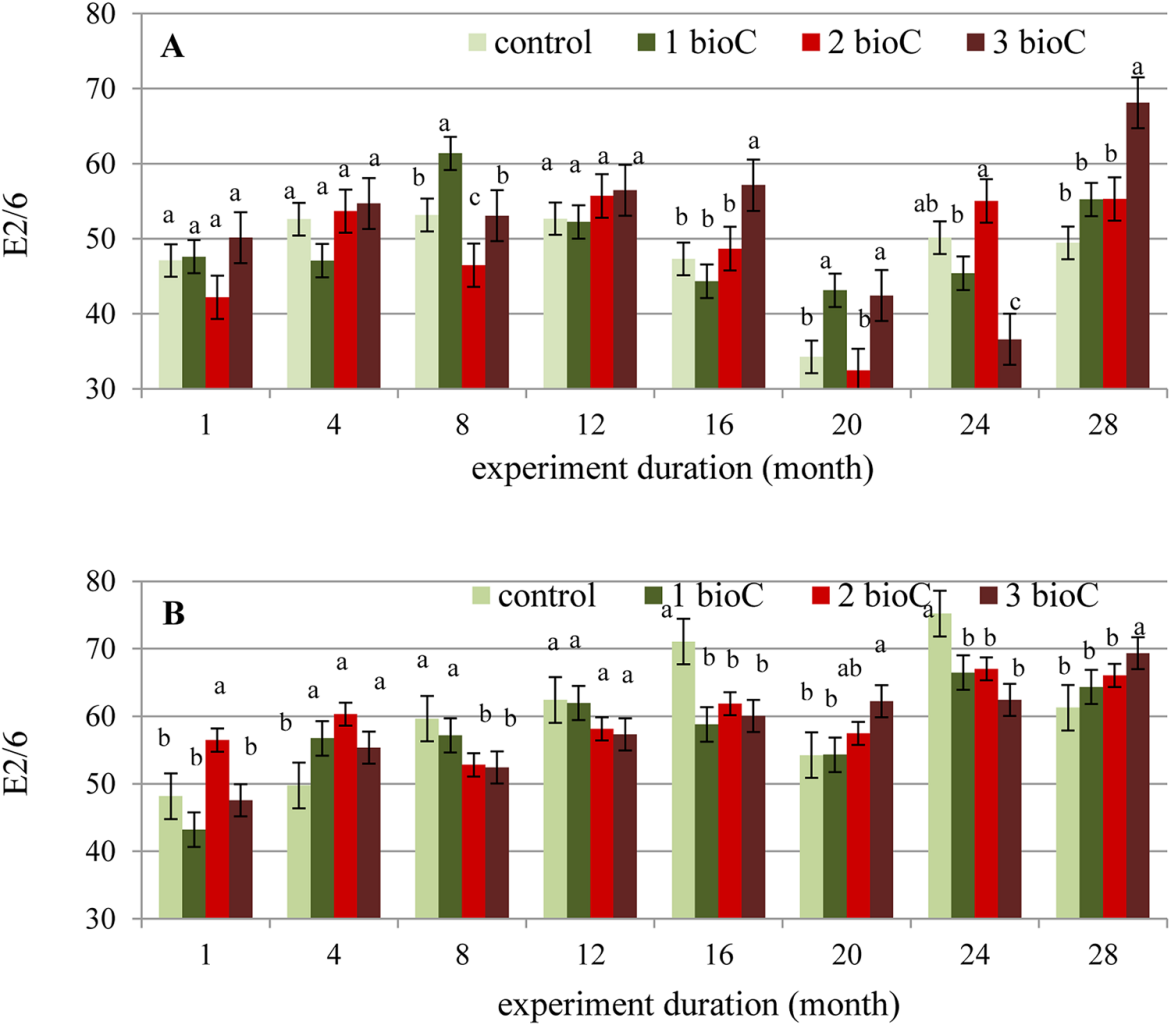
Changes in E_2/6_ values obtained for HAs of fallow (**A**) and grassland (**B**) amended with BioC (0, 1, 2, 3 kg m^−2^) as a function of time. Average values from 3 replicates in each term, ± standard deviation. Other letter designations indicate significant differences between values at α < 0.05.

The analyses of the HAs isolated from fallow and grassland amended with BioC showed changes in the structural properties of these compounds. The E_2/6_ parameter estimated from UV–Vis data was changing both under the influence of different BioC doses and during the 3 years of the experiment. However, it should be assumed that the observed changes were of a different nature for fallow (Fig. 5A) and for grassland (Fig. 5B), due to varied trends in the activity of BioC on the analysed soils.

In the case of fallow, the increase in BioC dose caused, for most analysed terms, a slight but insignificant increase in the E_2/6_ values of HAs. Additionally, over the 3 years of the experiment, sinusoidal changes in the E_2/6_ values were observed irrespective of BioC dose. In the case of grassland, the BioC dose did not clearly affect the E_2/6_ index. For this soil, however, a gradual increase in E_2/6_ value as a function of time was observed.

It should be noted that the value of E_2/6_ of the HAs isolated from BioC was relatively low in comparison to the values from soils (Table 1). Hence, most lignin-type compounds originated from the soil rather than from the BioC, and consequently, increasing the BioC doses did not cause an increase in the E_2/6_ of HAs. The periodic decreases and increases in the E_2/6_ values of HAs from the fallow observed during the time of the experiment most likely resulted from the higher exposure of OM contained in the fallow to the changing weather conditions (temperature, air humidity) as compared with that in the grassland. On the other hand, the continuous increase in E_2/6_ values of the grassland HAs as a function of the 3-year duration of the experiment may be related to the addition of further portions of lignin-type compounds, originated from fresh biomass, which were present on the grassland surface.

**Figure 6:**
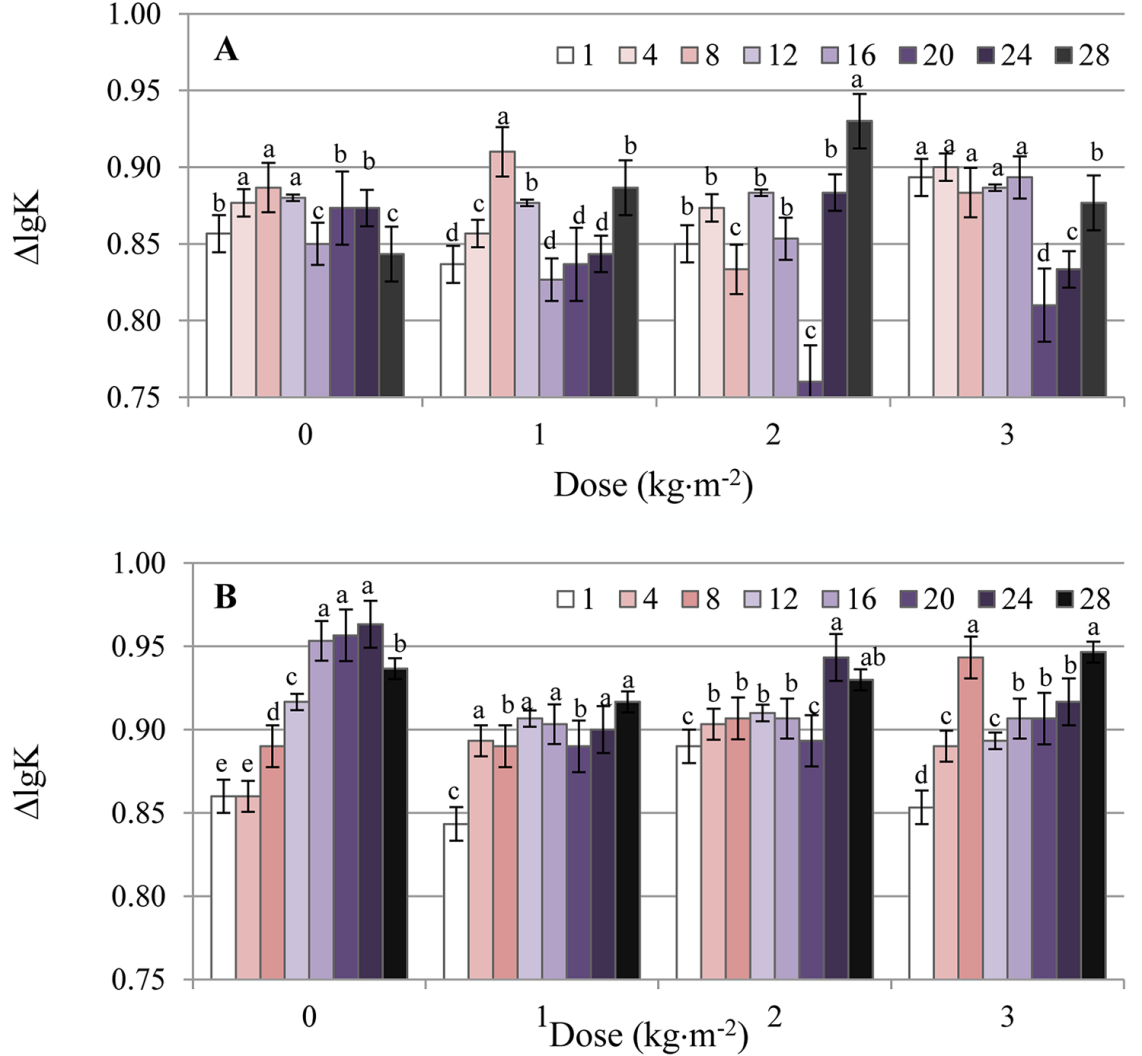
Changes in ΔlgK values obtained for HAs in fallow (**A**) and grassland (**B**) amended with BioC (0, 1, 2, 3 kg m^−2^) as a function of time (1, 4, 8, 12, 16, 20, 24, 28-months). Average values from 3 replicates in each term, ± standard deviation. Other letter designations indicate significant differences between values at α < 0.05.

Data obtained from spectrophotometric analyses allowed calculation of the ΔlgK parameter describing the humification degree of the HAs. Changes in the above parameter for HAs isolated from fallow and grassland amended with BioC are provided in Fig. 6A,B. The addition of increasing BioC doses did not clearly affect the value of ΔlgK of HAs originated from the fallow in relation to the control values for a specific term. No unambiguous effect of BioC was observed as a function of time. However, the addition of BioC to the grassland showed a significant effect on the humification degree of soil HAs. The humification process of the HAs in the control soil decreased over the duration of 3 years (ΔlgK increased from the first to the 28^th^ month of the experiment), while the BioC addition reduced the is decrease in degree of humification as a function of time, which was expressed in lower and more consistent ΔlgK values in months, as compared to the control soil.

An ambiguous effect of BioC on the humification degree of the HAs isolated from fallow could similarly be explained in the case of E_2/6_ (i.e. because during the experiment, fallow plots were maintained without plant cover and were exposed to varied weather conditions). Moreover, literature indicates that, the humification process of the OM is stimulated mainly by the presence of microorganisms and plant residues^59^. Therefore, the lack of plant cover suggests lower biological activity of the soil and, consequently, a lower intensity of humification processes. The reduction in ΔlgK (i.e. the increase in the humification processes) in relation to the control sample, as well as the stabilisation of ΔlgK values as a function of time after BioC supplementation, can be explained for grassland in two ways. Firstly, there is possibility that BioC may stimulate and accelerate the humification process of soil OM^41^. Moreover, BioC supplementation resulted in the direct introduction of strongly humified HAs (see ΔlgK of BioC, Table 1), which could also affect the observed decrease in ΔlgK values of grassland HAs. However, such an effect of BioC was not observed in the case of fallow, and we therefore assume that BioC added to grassland soil (rich in microorganisms and green biomass) may play an important role as a stimulator of microbial activity, and consequently, may intensify humification.

Indeed, humification processes are chemical and biochemical transformations, where microorganisms play a key role in the decomposition of OM into simpler organic structures. In these processes, the organic substrate is recolonised by microorganisms that degrade the residual sugars, cellulose, and hemicellulose, and increase the rate of polymerisation of the organic compounds to the humic substances^60^. Proliferated microbial population can produce more enzymes that result in proper humification^61^. In our studies, the beneficial effect of microorganisms in humification process could relate to better microorganism succession, which could be more effective on grassland than in fallow. In the case of BioC supplementation, decomposable and easily extractable BioC C could be utilised as a carbon source by bacterial and fungal populations for their growth^19^. This process may relate to enhanced mineralisation of organic carbon, but it could also contribute to the development of humus. In turn, highly stable BioC C can sorb organic molecules and promote their polymerisation to the new organic compounds^62^. Moreover, the adsorption of easily degradable compounds on the BioC surface can protect them from microbial decomposition^63^. The large surface area and well-developed pore network of our BioC^4^ could also positively affect soil microbial metabolism^53^ and could create excellent habitat conditions for the growth of bacteria and fungi. The progress of humification processes under the BioC and microbial influence which was observed in our experiments was similarly confirmed in previous study^64^. The authors of that research reported the highest increase in HA/FA ratio in the composting system with 100 g kg^−1^ bamboo BioC simultaneously indicating the strongest acceleration of humification under those conditions. Similarly, results of other studies^65^ on the effect of BioC on composting processes, proved the intense humification of organic substrate (more than 90% of HAs in alkali extract), as well as an increase in the ratio of HAs to fulvic acids.

**Figure 7:**
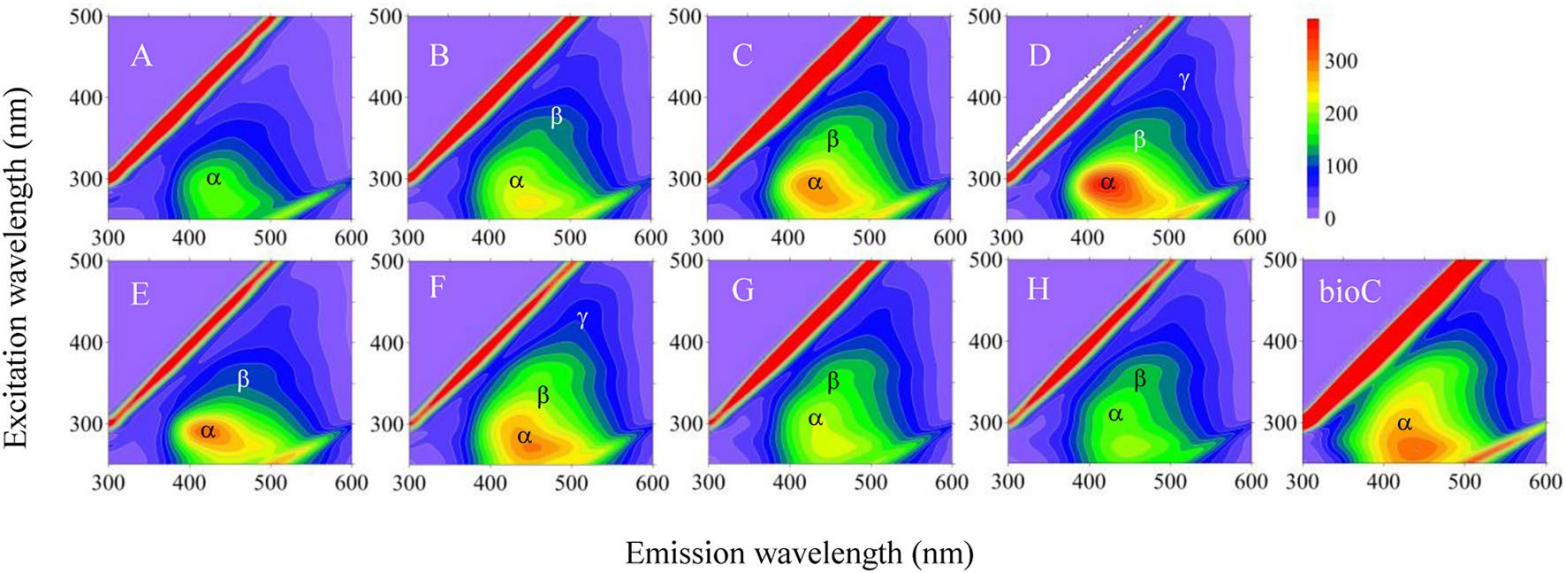
Excitation-emission matrices (EEM) for HAs obtained from: fallow without BioC in 1st (**A**) and 28th (**E**) month of field experiment; fallow amended with BioC (3 kg m^−2^) in 1st (**B**) and 28th (**F**) month; grassland without BioC in 1st (**C**) and 28th (**G**) month; grassland amended with BioC (3 kg m^−2^) in 1st (**D**) and 28th (**H**) month of field experiment; as well as HAs obtained from BioC. The figure was prepared with using Surfer software (Surfer, Golden Software, version 12.8.1009, https://www.goldensoftware.com/products/surfer).

The results of the fluorescence analysis supplemented the influence of BioC on the HA structure in terms of changes in fluorescent groups. The obtained EEM data showed the presence of one local maximum for fluorescence (marked as α)—the results are shown in Fig. 7 (Fig. 7A–D—1st month; Fig. 7E–H—28th month). The broadening of fluorescent bands was also observed in some regions (marked as β, γ, and ω), which suggests the presence of additional fluorophores. However, the β, γ, and ω peaks were concealed in some EEM spectra, which was probably caused by the overlapping signals of neighbouring fluorophores.

The α, β, and γ signals corresponded with the presence of various kinds of humic structures^66^. The activity of the α area was visible in all studied HA samples at excitation/emission wavelengths of 305–330 nm and 420–430 nm. According to the literature^67–69^, the α region was related to terrestrial humic-like substances derived from lignin and may reflect simple structural components of low molecular weight, low content of conjugated and aromatic groups, and low degree of humification. The highest fluorescence intensities (FIs) of this region were observed for HAs of grassland amended with BioC (3 kg m^−2^) during the first month of the field experiment (Fig. 7D). This fact also indicates that BioC provided OM containing a high number of electron-donating substituents (like hydroxyl/methoxyl groups) for these HAs^70^. The probable sources of HA-like components were considered as autochthonous, terrestrial or soil OM, or microbial processes^68^. HAs obtained from BioC were characterised by a relatively high FI signal of the α region, and consequently, BioC addition resulted in a higher FI signal during the first month of the experiment, in comparison with the soil without BioC (Fig. 7A–D). However, an opposite trend was observed in the last year of experiment. These results are consistent with results of another study^71^ in which the fluorescence intensity of the α region decreased in a submerged membrane bioreactor with pre-ozonation, suggesting the biodegradable DOM with fluorescence was gradually metabolised by microorganisms. The changes in the α region indicated an increase in the number of structures characterised by low molecular weight, low content of conjugated and aromatic groups, and low humification degree in the HA structure due to the BioC addition. The newly formed pool of low-molecular and weakly humified HAs was an extremely beneficial effect that confirmed our previous conclusions about the stimulating effect of BioC on the humification process in grassland (changes in ΔlgK). At the same time, it should be noted that together with such HAs, fulvic acids can be formed. This fraction is also characterised by a low molecular weight and a low degree of humification^70^. This could be important information for future research that includes the fulvic acid fraction. We suppose that this fraction may be important regarding humification products formed in a relatively short time. The β signals were placed at ex/em: 370–380 nm/480–490 nm, with the strongest signal for HAs obtained from the grassland during the first month of the experiment with and without the addition of BioC (Fig. 7C,D, respectively). The fluorescence of this region could be ascribed to the presence of moderately humified structures, simple phenols, coumarins, alkaloids, semiquinones, and hydroquinones^69,72^. There were no clear changes observed in the β region, so we can conclude that BioC did not deliver moderately humified structures to the soil’s HA structure. The HAs obtained from BioC and from fallow without the addition of BioC (see Fig. 7A) did not demonstrate a fluorescence area at the β sites. This signal could have overlapped with signals from other structures.

The γ area was observed for HAs obtained from grassland with BioC addition during the first year of experiment (Fig. 7D), as well as from fallow with BioC addition during the last year of the experiment (Fig. 7F). This signal was placed at ex/em: 430–445 nm/510 nm, which can be associated with the presence of unsaturated bonds, high molecular weight, high humification degree, and linearly condensed aromatic rings formed from lignin oxidation^73^. The FI of the discussed samples were similar, which could indicate a similar concentration of electron-withdrawing groups (like carbonyl groups). The BioC addition caused an increase in the number of highly humified structures in the HAs obtained from grassland during the first year of the experiment, and from fallow in the last year of the experiment.

## Materials and methods

**Figure 8:**
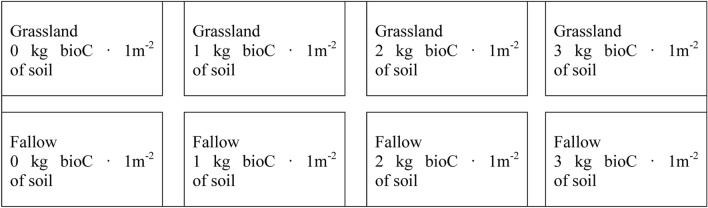
Scheme of field experiment.

The studies were conducted on a Haplic Luvisol (IUSS Working Group WRB)^74^ (51°15′ N; 22°35′ E) and included 4 fallow and 4 grassland subplots, each of 20 m^2^, with a separation of 1 m between the plots. Figure 8 provides a scheme of the field experiment.

The grassland was established at least 35 years ago and was managed by cutting the surface covered with various grass species, such as: *Festuca pratensis* Huds., *Dactylis glomerata* L., *Poa pratensis* L., and *Lolium perenne* L. The fallow surface had been left unseeded after being tilled to a depth of 15 cm and harrowed for 10 years prior to the experiment. During the research, the fallow plots were maintained without plants. All plots, before and during the experiment, were fenced to ensure that animals were not able to enter.

The BioC used in the experiment was obtained via wood waste (coniferous and deciduous) pyrolysis at 650 °C (Fluid S.A.); the pyrolysis time was 15 min, and the heating rate was approximately 3 K s^−1^. The dry product was uniformly applied to the surface of the subplots at densities of 0 (control), 1, 2, and 3 kg m^−2^ in June of 2013. The BioC doses were selected based on our previous studies on the impact of BioC on the physicochemical properties of Haplic Luvisol soil under different land use^4^. In the case of fallow, BioC was mixed to a depth of 0–15 cm (surface layer) using a rototiller, and it was left on the surface in the case of grassland^75,76^. To avoid BioC loss, the plots were initially wetted. Soil samples were collected (in triplicate) from the surface layer (0–15 cm) from each fallow and grassland plot, during the summer, autumn, and spring periods (July, November, and March, respectively) during the 3-year experiment: two times in the first year (starting from July—the first month of the experiment), three times in the second year, and three times in the third year (the last one in November which was the 28th month of the experiment). The collected material was dried at 25 °C, sieved through a 1 mm sieve, and then grounded.

### BioC and soil characterisation

Selected physicochemical and chemical properties were determined for soil and BioC characterisation. The density (d) was determined using the pycnometric method (Ultrapycnometer 1000, Quantachrome, Graz, Austria); the pH was measured in H_2_O and 0.01 mol·dm^−3^ CaCl_2_ solution (Multifunction pH-meter CX-505, Elmetron, Zabrze, Poland). The ash content (A) was determined as the loss of BioC calcination in a muffle furnace (FCF 12 SP, Czylok, Jastrzębie-Zdrój, Poland) at 550° C for 5.5 h. The organic carbon content (C_org_) was analysed using a TOC analyser (Multi N/C 2000, Analityk Jena, Jena, Germany). The negative surface charge (Q) was calculated at pH 9.0 from potentiometric titration curves recorded for aqueous suspensions of soil or BioC (5 g kg^−1^) titrated with 0.1 mol·dm^−3^ NaOH (based on 1 mol dm^−3^ NaCl) in a pH range of 3.0 to 10.0 using the automatic titrator TitroLine 7000 (SI Analytics, Mainz, Germany)^26^.

### Analysis of BioC influence on soil OM and HAs

The effect of BioC addition on the OM of the soils was investigated with an analysis of the changes in C_org_ content in the soils using the same method as for the control soil characterisation. The distribution functions of the apparent dissociation constants of the functional groups were determined for the OM in soils amended with BioC based on the potentiometric titration curves obtained for the mixture of soil with BioC in the pH range of 3.0–10.0 using the same conditions and calculations as for the characterisation of the initial materials.

HAs were isolated from the BioC, control soil, and soil amended with BioC using an alkaline extraction method proposed by the International Humic Substances Society^77^. The changes in the HA organic carbon concentration (C_orgHA_) were analysed in the HA alkaline extracts using a TOC analyser (Multi N/C 2000, Analityk Jena, Jena, Germany). The UV–Vis spectra were recorded for HA solutions (concentration = 40 mg dm^−3^) in the wavelength range of 200–800 nm (UV–Vis spectrophotometer V-530, Jasco INC, Tokyo, Japan). From the obtained spectral data, the ΔlgK parameter^78^ (describing the HA humification degree) and E_2/6_ coefficient (describing the ratio of lignin-type compounds to the content of substances with a high degree of humification) were calculated as follows:

1 $$ \Delta lgK = lgA_{400nm} - lgA_{600nm} $$

2 $$ E_{2/6} = A_{280nm} /A_{665nm} $$

where A_x_ is the absorbance measured at a specific wavelength.

The absorption spectra of HAs were determined in dissolved 0.025 mol dm^−3^ NaOH, and the absorption curves were promptly drawn in the region of 220–700 nm^78^. These curves were expressed as lg K-λ curves of 10 g dm^−3^ HA solution, where K and λ were the optical density and wavelength, respectively.

The changes in Q_HAs_ were determined for HAs of soils amended with BioC based on the potentiometric curves in the pH range of 3.0–9.0 using the same titration conditions as in the case of the characterisation of the initial materials’. The values of ΔlgK, E_2/6_ as well as the Q_HAs_ for the HAs of the control soils and for the BioC were attached to the data describing the research materials.

The HA solutions for the fluorescence measurements were prepared by dissolving 40 mg of each lyophilised sample in 1 dm^3^ of deionised water. The pH of the solutions were adjusted to 7.5 ± 0.1 using 0.1 mol dm^−3^ NaOH. The emission-excitation fluorescence 3-D matrices (EEM) were recorded with a scan speed of 30,000 nm min^−1^ using the Hitachi F-7000 FL luminescence spectrometer (Hitachi, Tokyo, Japan). The emission wavelength was scanned from 300 to 600 nm, the excitation wavelength was raised sequentially in 5 nm steps in the range of 250–500 nm. The analyses were preceded by fluorescence calibration using quinine sulphate at λ_ex_ = 350 nm and λ_em_ = 450 nm. Spectral correction was performed using rhodamine B. The EEM data were processed into contour maps using Surfer software (Golden Software Inc., Golden, CO, USA)^70^.

Statistical analysis was performed using Statistica 12.0 (StatSoft Inc., Tulsa, USA): a one-way analysis of variance (ANOVA) and a post hoc analysis (HSD Tuckey test, α = 0.05).

## Conclusions

The results obtained suggested that the BioC application seemed to have a subtle influence on soil characteristics, leading to changes in the OM, including HAs. The addition of BioC had various influences on differently cultivated soils—fallow and grassland.

The addition of increasing BioC doses caused an increase in C_org_ content which ranged for different months from 3 to 20% and from 15 to 25% as compared to control values in the fallow and grassland, respectively. The C_org_ content in both soils after the addition of BioC remained constant throughout entire 3-year experiment, indicating the high stability of organic compounds introduced with BioC.

BioC addition to the fallow did not significantly influence the parameter for the content of lignin-type compounds (E_2/6_) for HAs originated from this soil. Similarly, the addition of BioC to the fallow did not change the humification degree (ΔlgK) of this soil’s HAs. In the case of grassland, a gradual increase in the E_2/6_ values of the HAs was observed during the 3-year experiment, indicating an increase of lignin-type compounds in the structure of the soil’s HAs. The application of BioC to the grassland stimulated and accelerated the humification process of this soil’s HAs. Moreover, the content of HAs in both soils increased significantly with an increase in the BioC dose at each term of the experiment, suggesting an increase in soil sorption capacity.

Based on the EEM data analysis, we can conclude that BioC addition to the soil caused an increase in the number of structures characterised by low molecular weight and a low degree of humification. Furthermore, its addition caused an increase in the structures characterised by a high degree of humification in the HA structures obtained from grassland in the first year of the experiment and from fallow in the last year of the experiment.

The results of this study support the thesis that the addition of BioC to degraded soils may improve their fertility.
